# High-dimensional randomization-based inference capitalizing on classical design and modern computing

**DOI:** 10.1007/s41237-022-00183-x

**Published:** 2022-09-28

**Authors:** Marie-Abele C. Bind, D. B. Rubin

**Affiliations:** 1 Biostatistics Center, Massachusetts General Hospital, 50, Staniford Street, Boston, MA 02114 USA; 2 Yau Center for Mathematical Sciences, Tsinghua University, Beijing, China; 3 Department of Statistical Science, Fox School of Business, Temple University, Philadelphia, PA USA

**Keywords:** Big data, Causal inference, Randomization-based tests, Sharp null hypotheses, Fisherian inference, Fisher-exact *p*-value, Test statistic, Randomized crossover experiment, Large *P*, Small *N* data, Ozone, Air pollution, Epigenetics

## Abstract

A common complication that can arise with analyses of high-dimensional data is the repeated use of hypothesis tests. A second complication, especially with small samples, is the reliance on asymptotic *p*-values. Our proposed approach for addressing both complications uses a scientifically motivated scalar summary statistic, and although not entirely novel, seems rarely used. The method is illustrated using a crossover study of seventeen participants examining the effect of exposure to ozone versus clean air on the DNA methylome, where the multivariate outcome involved 484,531 genomic locations. Our proposed test yields a single null randomization distribution, and thus a single Fisher-exact *p*-value that is statistically valid whatever the structure of the data. However, the relevance and power of the resultant test requires the careful a priori selection of a single test statistic. The common practice using asymptotic *p*-values or meaningless thresholds for “significance” is inapposite in general.

## Introduction

Published data analyses from some fields rich with data, such as genomics and epigenomics, often fail to report valid and informative *p*-values when faced with many tests (e.g., Zeilinger et al. 2013; Morozova et al. 2015). Instead, some reported alternatives aim to “adjust” for a plethora of calculated *p*-values, using crudely applied Bonferroni adjustments (1936), which are typically grossly conservative (Perneger 1998).

In contrast, we describe a procedure for generating a single null randomization distribution for a large set of sharp null hypotheses, and thus a single Fisher-exact *p*-value. To be helpful, the resulting *p*-value depends critically on a priori choosing a revealing scalar test statistic, that is sensitive to scientifically interesting departures from the sharp null hypothesis, which is typically that there is absolutely no effect of treatments being studied on any of the outcomes. Making this choice of test statistic generally makes demands on the researcher to think carefully before attacking the data, a task which is explored in the context of our example. This approach also relies on modern computing environments to implement, but the demands on the researcher to *think* before computing is more critical. This is especially true in the current environment of ever-increasing computational power, which allows many superficial exploratory analyses. Such analyses, although apposite to conduct, should be postponed until after confirmatory analyses have been completed. We illustrate the essential idea in the context of a randomized crossover experiment, where we compare two treatment conditions on a multi-component epigenetic outcome measured at almost half a million epigenomic locations.

## Background

### Epidemiological context

Epidemiological studies in humans have reported associations of ozone exposure with mortality (Bell et al. 2004; Jerrett et al. 2009; Turner et al. 2016), as well as with epigenetic marks (e.g., Bind et al. 2014), but better support for causal relationships comes from studies with controlled exposures (Devlin et al. 2012). Suggestive results have been seen in a randomized controlled study with rats (Miller et al. 2018). However, there is the desire for studies exploring evidence for causality between ozone exposure and the DNA methylome in humans using randomized experiments.

### The randomized crossover experiment

The epigenetic study used here has been previously described (Bind et al. 2020). Briefly, a total of seventeen healthy young adults (indexed by $i=1,\ldots ,N$) were exposed on two occasions, separated by at least thirteen days, once to clean air and once to ozone, randomly ordered (see Table 1), for two hours while exercising intermittently. We denote the $i\mathrm{th}$ participant’s treatment assignments at periods $j=1$ and $j=2$ by the two-component row vector $W_i=(W_{i,j=1}, W_{i,j=2})$.

**Table 1:** Notation for the randomized crossover experiment

Number of participants	$W_i$	Visit $j=1$	Visit $j=2$
7	$(W_{i,j=1}=0, W_{i,j=2}=1)$	Exposed to clean air	Exposed to ozone
10	$(W_{i,j=1}=1, W_{i,j=2}=0)$	Exposed to ozone	Exposed to clean air

After each exposure, each participant underwent bronchoscopy during which epithelial cells were removed and used to measure DNA methylation on each of 484,531 CpG sites along the genome with the Illumina 450K platform.^1^ For each participant, the scalar outcome $Y=$ “DNA methylation on CpG sites” is defined as the proportion of methylated cytosines over all methylated and unmethylated cytosines.

In this experiment, the exposure factor thus has two levels, clean air and ozone, and each participant is randomized at period $j=1$ to one or the other treatment level, and the exposure level at period $j=2$ differs from the exposure level at period $j=1$ for all units. A randomized crossover experiment is a fractional design of a more general randomized experiment, where both exposure factors have two levels, clean air and ozone, are randomized at period $j=1$ and at period $j=2$.

The objective is to learn whether ozone exposure has an effect on the DNA methylome. First step is to understand DNA methylation under clean air, and then to anticipate deviations under ozone exposure.

### The DNA methylome under clean air: a set of dependent outcomes

Common analyses of the DNA methylome typically assume its myriad components are independent.

**Fig. 1:**
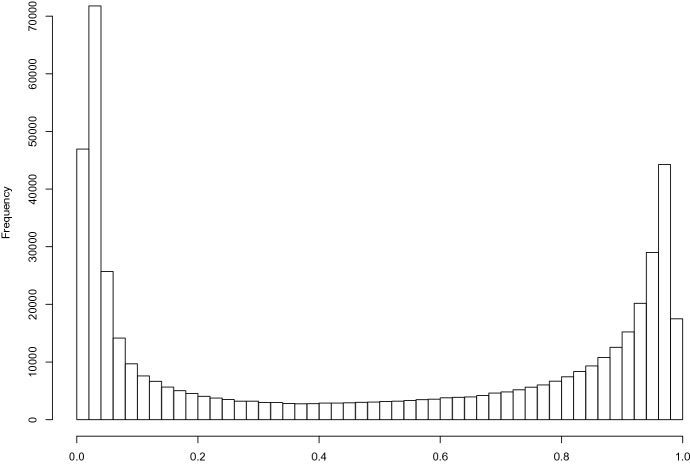
Histogram of the $K=484{,}531$ sample means of methylation under clean air averaged across seventeen participants at each CpG site. The horizontal axis indicates the methylation values, which is the proportion of probe signal intensities that are methylated. Notice the obvious bimodal distribution, which supports previous conclusions from the literature that under clean air, most sites are either nearly fully methylated or unmethylated

DNA methylation appears to act as a “switch” that controls gene expression because its distribution across regions is bimodal with most regions either highly methylated or essentially unmethylated (Bennett and Hasty 2007). This feature is also seen in our data: Fig. 1 presents the empirical density of the sample means of methylation under clean air (whether at first or second period) averaged over the seventeen participants at each of the 484,531 CpG sites (which are indexed by $k \in \{1, \ldots , 484{,}531\}$ and ordered by chromosome and genomic location). This figure reveals a bimodal distribution of means over seventeen participants across CpG sites, which is also seen at the participant-level.

**Fig. 2:**
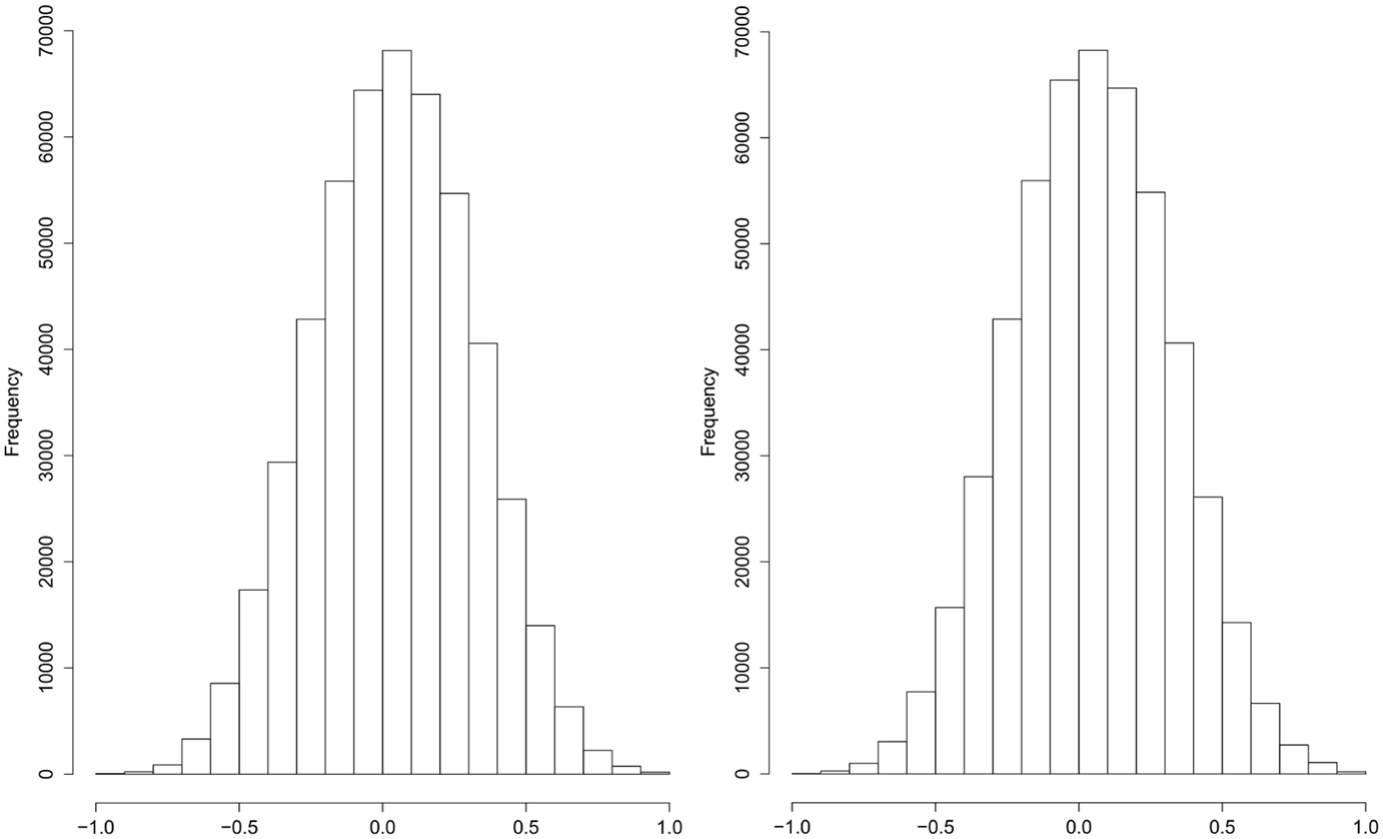
Histogram of the correlations between the first 1000 adjacent CpG sites (left) and between 1000 randomly drawn CpG sites (right) under clean air

With such a high-dimensional outcome, the typical hope is to capitalize on some independence or at least some exchangeability somewhere. However, high-dimensional epigenomic outcomes are relatively complicated and consist of possibly correlated DNA methylation measurements at the 484,531 CpG sites. To explore this possible correlational structure, Fig. 2 presents the distributions of the upper triangle of the matrix of all pairwise correlations between: (1) measurements of the first 1000 “adjacent” CpG sites on chromosome 1 measured by the 450K array under clean air (left panel), and (2) 1000 randomly drawn CpG sites under clean air (right panel). The number of elements in the upper triangle of the correlation matrices is thus $\frac{1000*999}{2}=499{,}500$.

**Fig. 3:**
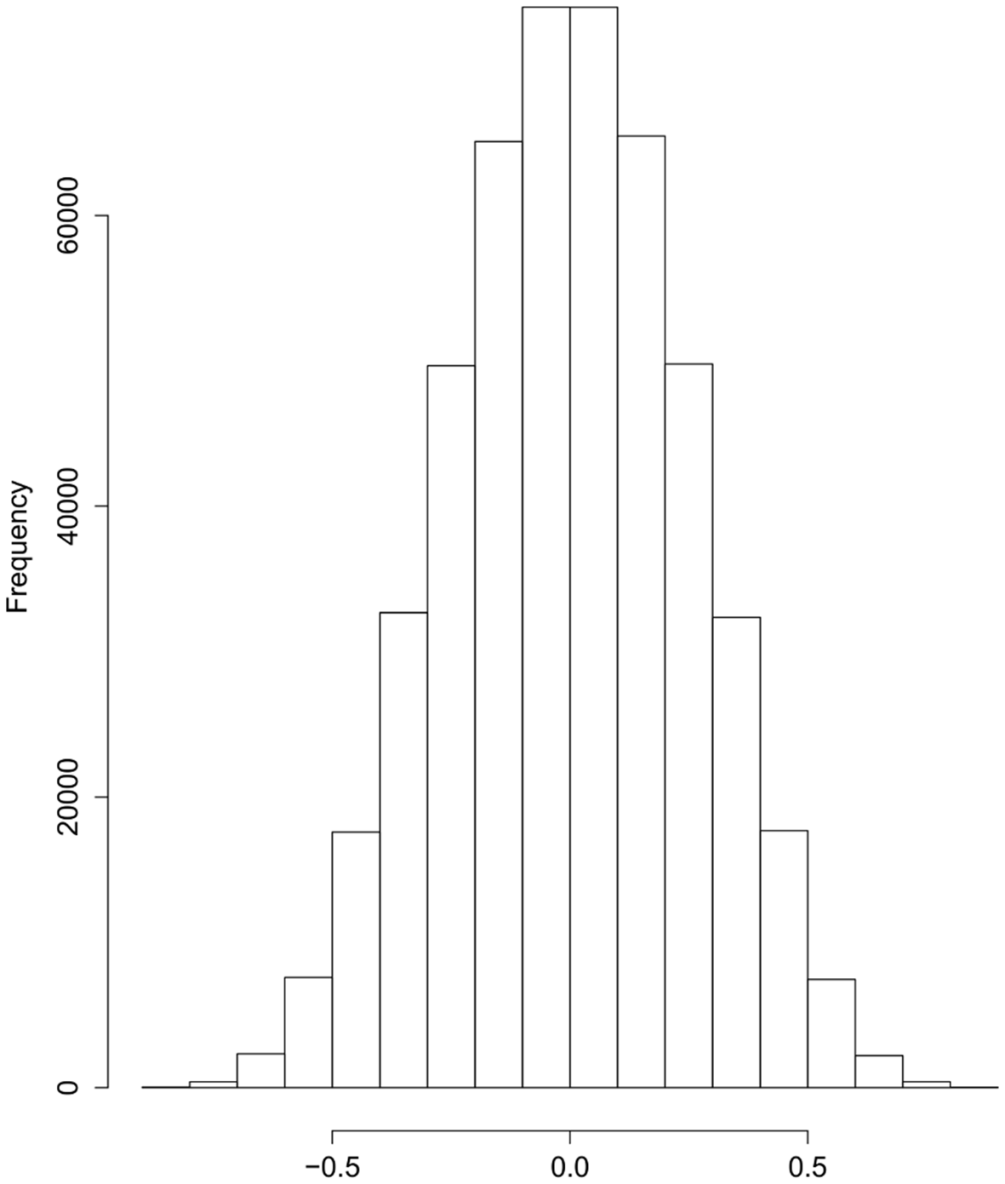
Histogram of 249,750 randomly drawn variables distributed as $\sqrt{Beta\left( \frac{1}{2}, \frac{17-2}{2}\right) }$ and 249,750 independently randomly drawn variables distributed as $-\sqrt{Beta\left( \frac{1}{2}, \frac{17-2}{2}\right) }$

For comparison with simple randomness, Fig. 3 presents the simulated distribution under the null hypothesis of no correlation between 1000 normal random variables: We drew 499,500 independent variables such that their squared values follow a ${Beta(\frac{1}{2}, \frac{N-2}{2})}$ distribution, which is the null distribution of the estimated squared-correlation coefficient between two independent normally distributed random variables.

**Fig. 4:**
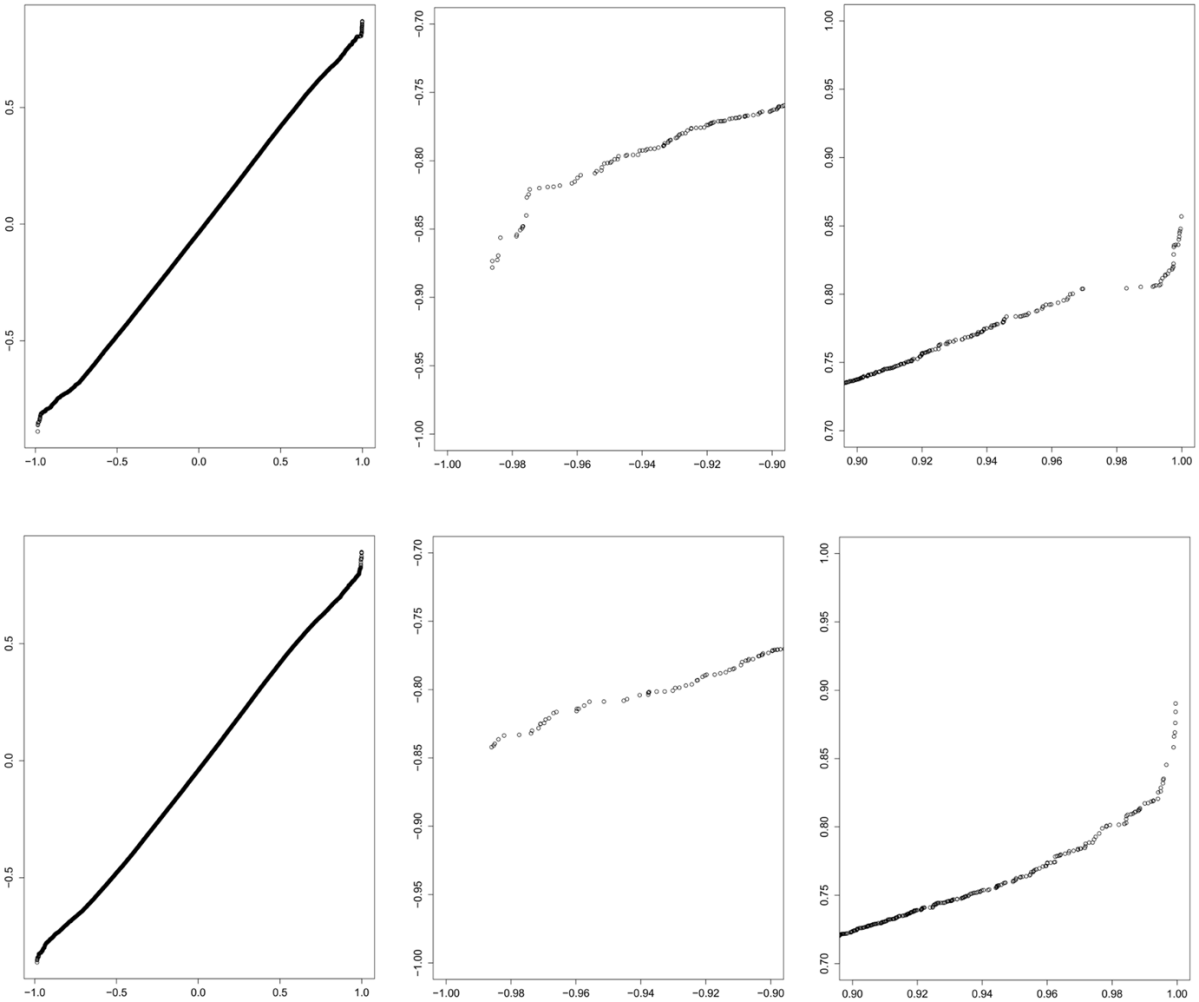
QQ-plots comparing the randomly drawn 499,500 variables in Fig. 3 to the elements in the upper triangle of the matrix of the pairwise correlations of: (1) the first 1000 “adjacent” CpG sites on chromosome 1 under clean air (top left) followed by zooms of the bottom-left (top middle) and top-right corners (top right), and (2) of the 1000 drawn at random CpG sites under clean air (bottom left) and zooms of the bottom-left (bottom middle) and top-right corners (bottom right). These zooms reveal visually-obvious departures from the theoretical distribution under normal exchangeability

Based on Figs. 2 and 3, it is difficult to assess visually whether levels of DNA methylation on CpG sites are uncorrelated in our data set. It appears that the right tail of the empirical correlation distributions (Fig. 2) may have longer tails than the distribution assuming normality and independence, which is displayed in Fig. 3. Figure 4 presents Q-Q plots comparing the correlations under normality and independence, i.e., $\pm \sqrt{Beta(\frac{1}{2}, \frac{17-2}{2})}$ to the empirical correlations between: (1) the first 1000 adjacent CpG sites on chromosome 1 under clean air, and (2) the 1000 randomly drawn CpG sites under clean air, respectively. We enlarge the plots at both extremes to focus on the tails. The extreme tails do not follow a 45$^\circ$ line, suggesting that, even under clean air, there are more extreme correlations than expected under normality and independence, suggesting that the joint assumption, normality and independence, is suspect for those data, and thus pursuing analyses based on the usual normal and independence assumptions could be deceptive.

### Typical Bonferroni correction applied to the Fisher exact *p*-value

In biomedical research, it is common to have studies like this one, with a small number of participants compared to the number of outcomes; such data sets are sometimes referred as “small N, big P data”. In such settings, it is common to control the family-wise error rate (FWER) of statistical tests, the probability of making one or more false “discoveries” (also called false positives or type I errors). Let $\alpha$ be the statistical significance level for each of the *K* statistical tests we perform of the sharp null hypothesis. The Bonferroni “correction” (Bonferroni 1936) states that we should declare as “significant”, or more accurately possibly “worth further scrutiny” (Edgeworth 1885; Fisher 1925; Wasserstein et al. 2019), only those *p*-values that are less than or equal to $\frac{\alpha }{K}$, and that doing so will control the FWER to be less or equal to $\alpha$.

For example, in a randomized crossover experiment with N participants and two treatment levels (i.e., either $W_i=(0,1)$ or $W_i=(1,0)$ with our notation), the smallest Fisher-exact *p*-value that can be obtained is equal to the inverse of the total number of possible randomizations, that is, for a 50–50 Bernoulli assignment mechanism with *N* units, $\frac{1}{2^N}$. In our example with $N=17$ individuals, even assuming a Bernoulli assignment mechanism (which maximizes the number of possible randomizations and therefore minimizes the possible Fisher-exact *p*-value), the minimal Fisher-exact *p*-value that can be obtained from one test is equal to $\frac{1}{2^{17}} = \frac{1}{131,072} \approx 7.63\times 10^{-6}$. The associated Bonferroni “correction factor” in this example with $K=484{,}531$ tests is equal to $\frac{0.05}{484{,}531} \approx 1.03\times 10^{-7}$, which reveals that, a priori, the Bonferroni adjustment offers zero power in this experiment because all such “adjusted” *p*-values exceed 1! Therefore, this commonly-used adjustment method has no practical value in situations like our example. In a similar crossover study with $N=10$ individuals (Zhong et al. 2017), the minimal attainable Fisher-exact *p*-value that can be obtained (assuming a Bernoulli assignment mechanism), would be equal to $\frac{1}{2^{10}} = \frac{1}{1,024} \approx 10^{-3}$. Because all “Bonferroni-adjusted” *p*-values exceeded 1, the authors lowered the significance threshold to an arbitrarily value of $10^{-4}$, which was inapposite, not only because of the FWER, but also because the chosen threshold was below the minimum attainable Fisher-exact *p*-value. There must be a better way for us to attack this problem!

## Our proposed method

### Notation

The following sections may appear overly tedious with respect to their notations. However, we believe that the statistical issues described here have been imprecisely defined, especially in crossover studies.

#### Potential outcomes and assumptions

As in Table 1, let ${W_i}=(W_{i,j=1}, W_{i,j=2})$ denote the assignment of exposures for participant *i*, $i \in \{1,\ldots ,N=17\}$. After exposure to a treatment at each period *j*, for each participant *i*, a multivariate outcome measurement with dimension K, generically denoted $Y_{i,j}=(Y_{i,j}^1, \ldots , Y_{i,j}^k, \ldots , Y_{i,j}^K)$, is taken. We use the notation of potential outcomes to define the possible observed values in this experiment (Neyman 1990; Rubin 1974, 1990). Under the stable unit treatment value assumption (SUTVA) (Rubin 1980), the values of the four *K*-dimensional potential outcome measurements that could be observed for participant *i* assigned to ${W}_i=(W_{i,j=1}, W_{i,j=2})$ are: $Y_{i,j=1}(W_{i,j=1}=0)$, $Y_{i,j=1}(W_{i,j=1}=1)$, $Y_{i,j=2}(W_{i,j=1}=0, W_{i,j=2}=1)$, and $Y_{i,j=2}(W_{i,j=1}=1, W_{i,j=2}=0)$, as displayed in Table 2.

**Table 2:** Science table of all possible outcomes of the randomized crossover experiment

Participant	$W_i$	$Y_{i,j=1}\,(W_{i,j=1}=0)$	$Y_{i,j=1}\,(W_{i,j=1}=1)$	$Y_{i,j=2}\,(W_{i,j=1}=0, W_{i,j=2}=1)$	$Y_{i,j=2}\,(W_{i,j=1}=1, W_{i,j=2}=0)$
1	$W_1$	$Y_{1,j=1}\,(W_{1,j=1}=0)$	$Y_{1,j=1}\,(W_{1,j=1}=1)$	$Y_{1,j=2}\,(W_{1,j=1}=0, W_{1,j=2}=1)$	$Y_{1,j=2}\,(W_{1,j=1}=1, W_{1,j=2}=0)$
.	.	.	.	.	.
*N*	$W_N$	$Y_{N,j=1}\,(W_{N,j=1}=0)$	$Y_{N,j=1}\,(W_{N,j=1}=1)$	$Y_{N,j=2}\,(W_{N,j=1}=0, W_{N,j=2}=1)$	$Y_{N,j=2}\,(W_{N,j=1}=1, W_{N,j=2}=0)$

Note that all *Y* values are vectors of dimension *K*

#### Causal estimands

Possible causal estimands of interest include the unit-level causal effect of assignment to ozone versus assignment to clean air at period *j* on the *K*-dimensional outcome:

$$\begin{aligned} \tau _{i,j=1}=Y_{i,j=1}(W_{i,j=1}=1)-Y_{i,j=1}(W_{i,j=1}=0) \end{aligned}$$

and

$$\begin{aligned} \tau _{i,j=2}=Y_{i,j=2}(W_{i,j=1}=0, W_{i,j=2}=1)-Y_{i,j=2}(W_{i,j=1}=1, W_{i,j=2}=0). \end{aligned}$$

The *K*-dimensional participant-level, period averaged, causal effect of the exposure to ozone vs. exposure to clean air on the high-dimensional outcome is $\tau _i=\frac{\tau _{i,j=1}+\tau _{i,j=2}}{2}$, and the grand average causal effect across participants is $\tau = \frac{1}{17} \sum _{i=1}^{17} \tau _i$. Of course, none of these causal estimands can be directly observed (Rubin 1978; Holland 1986).

Here, for $\tau _{i,j=2}$ to be an easily-interpretable estimand, we assume that the time between periods $j=1$ and $j=2$ is adequate to “wash out” the possible effect of the ozone exposure applied at period $j=1$ on the outcome measured at period $j=2$, i.e., $Y_{i,j=2}(W_{i,j=1}=1, W_{i,j=2}=0) = Y_{i,j=2}(W_{i,j=1}=0, W_{i,j=2}=0)$. This assumption has been commonly referred in the experimental design literature as the “no carry-over effect” assumption. Note that, for easy interpretation, we implicitly assume no “placebo effect” for each unit, which is plausible with our data, because the participants were blinded to their exposures.

In a randomized crossover experiment with the assumptions of no carry-over and no time effects, other participant-level contrasts of potential outcomes could be of scientific interest, such as $\tau _i^{CA,OZ}=Y_{i,j=2}(W_{i,j=1}=0, W_{i,j=2}=1) - Y_{i,j=1}(W_{i,j=1}=0)$ for participants exposed first to clean air, and $\tau _i^{OZ,CA}=Y_{i,j=1}(W_{i,j=1}=1) - Y_{i,j=2}(W_{i,j=1}=1, W_{i,j=2}=0)$ for participants exposed first to ozone. We will refer to these seventeen participant-level estimands of ozone exposure “minus” clean air as $\tau _i^\prime =\frac{\tau _i^{CA,OZ}+\tau _i^{OZ,CA}}{2}$, and their average across participants as $\tau ^\prime$.

#### Observed outcomes

Each *K*-dimensional unit-level observed outcome measured for participant *i* at period *j*, $y^{\mathrm{obs}}_{i,j}$, is a function of the treatment assignment $W_{i,j}$ and the potential outcomes at period *j*:

$$\begin{aligned} y^{\mathrm{obs}}_{i,j=1}=Y_{i,j=1}(W_{i,j=1}=0) * (1-W_{i,j=1}) + Y_{i,j=1}(W_{i,j=1}=1) * W_{i,j=1} \end{aligned}$$

and

$$\begin{aligned}&y^{\mathrm{obs}}_{i,j=2}=Y_{i,j=2}(W_{i,j=1}=0, W_{i,j=2}=1) * W_{i,j=2} \\&\quad + \ Y_{i,j=2}(W_{i,j=1}=1, W_{i,j=2}=0) *(1-W_{i,j=2}). \end{aligned}$$

Similarly, we define the participant-level observed outcome under clean air as:

$$\begin{aligned} y^{\mathrm{obs}}_{CA,i} = Y_{i,j=1}(W_{i,j=1}=0) * (1-W_{i,j=1}) + Y_{i,j=2}(W_{i,j=1}=1, W_{i,j=2}=0) * (1-W_{i,j=2}). \end{aligned}$$

The observed methylation mean under clean air at site *k* and the estimated standard deviation under clean air at site *k* are $\hat{\mu }_{CA}^k=\frac{1}{N}\sum _{i=1}^{N} y^{\mathrm{obs},k}_{CA,i}$ and $\hat{\sigma }_{CA}^k=\sqrt{\frac{1}{N-1}\sum _{i=1}^{N} (y^{\mathrm{obs},k}_{CA,i}-\hat{\mu }_{CA}^k)^2}$), respectively. The observed participant-level outcome at site *k*, $y^{\mathrm{obs},k}_{i}$, can be expressed as $(y^{\mathrm{obs},k}_{i,j=1}, y^{\mathrm{obs},k}_{i,j=2})$ or as $(y^{\mathrm{obs},k}_{CA,i}, y^{\mathrm{obs},k}_{OZ,i})$ in obvious, but not identical notations.

### Fisher’s sharp null hypothesis: exposure to ozone versus clean air has no empirical consequences

#### Naive sharp null hypothesis

The Fisher sharp null hypothesis ($H_{00}$) in this setting asserts that, for each participant *i*, there is no effect of exposure assigned at any period on any of the vector outcomes ${Y_{i,j}}$ measured at any period *j*, that is^2^:

$$\begin{aligned}&Y_{i,j=1}(W_{i,j=1}=0) = Y_{i,j=1}(W_{i,j=1}=1)\\&\quad \text {and } Y_{i,j=2}(W_{i,j=1}=0, W_{i,j=2}=1) = Y_{i,j=2}(W_{i,j=1}=1, W_{i,j=2}=0).^2 \end{aligned}$$

#### Tested sharp null hypothesis

The sharp null hypothesis that we are actually testing in this randomized crossover experiment contains two additional statements, i.e., there are no time and no carry-over effects. If we obtain evidence against the sharp null hypothesis, we would have to consider both additional assumptions, because such evidence is formally evidence against any aspect of the null hypothesis.

### Construction of a summarizing test statistic

To make each component of the K-component outcome comparable, we scale the $k^{\text {th}}$ component of the unit-level outcome measured at period *j*, $y^{\mathrm{obs},k}_{i,j}$, using the estimated methylation mean under clean air, $\hat{\mu }_{CA}$, and the estimated standard deviation under clean air $\hat{\sigma }_{CA}$, both described previously:

$$\begin{aligned} y^{\mathrm{obs},k}_{\mathrm{scaled}, i,j}=\frac{y^{\mathrm{obs},k}_{i,j}-\hat{\mu }_{CA}^k}{\hat{\sigma }_{CA}^k}. \end{aligned}$$

Notice that $y^{\mathrm{obs},k}_{\mathrm{scaled}, i,j}$ is a function of the clean air measurements for all units, a function of the observed randomization, and therefore, not a purely scientific estimand.

Assuming no carry-over and no time effects, the $k^{\text {th}}$ component of $\tau ^\prime$ can be estimated by regressing each vector of 34 unit-level observed outcomes on each participant’s treatment level at period *j*, thereby leaving us with $K=484{,}531$ estimated regression coefficients. Here, because the dimension of the outcome Y is large compared to the number of units, to conduct a randomization-based test, we need a scalar test statistic. We consider what is often called “regularization” approaches.

We choose a test statistic that reflects a fairly-popular approach used in biomedical research (e.g., Suchting et al. 2019), defined by the following two-step procedure:

**Step 1**: Let us consider a “backward and unnatural” logistic regression using 34 units (i.e., two time points per participant), which is sometimes referred as “reverse regression” (Conway and Roberts 1983):

$$\begin{aligned} \hbox {logit}[P(W_{i,j}=1)]=\beta _0 + \sum \limits _{k=1}^{484{,}531} \beta _k \, y^{\mathrm{obs},k}_{i,j,\mathrm{scaled}}. \end{aligned}$$

Our use of this statistic is not advocating the use of the “reverse regression” approach for estimating causal effects on an outcome, but rather simply illustrating how to construct a possibly useful scalar test statistic using a popular way of examining treatment-outcome associations in high-dimensional settings. Notice that here, this “backward” logistic regression is only used for defining our test statistic. Other approaches for choosing test statistics, for instance, based on subject-matter knowledge, are likely to be at least as scientifically revealing. The main motivation for this choice is to shrink the estimated regression coefficients $\hat{\beta _k}$ using a Lasso-type of regularization technique.

Specifically, we consider the generalized Elastic-Net regularization (Friedman et al. 2010), which recently has become a popular penalization strategy (e.g., Suchting et al. 2019). This regularization combines Lasso-type (i.e., by adding a first penalty term $\lambda _1 \sum \limits _{k=1}^{484{,}531} |\beta _k|$), and Ridge-type (i.e., by adding a second penalty term $\lambda _2 \sum \limits _{k=1}^{484{,}531} | \beta _k |^2$) penalizations, where $\lambda _1$ and $\lambda _2$ are two tuning parameters. As is sometimes recommended, we choose the tuning parameters that minimize the Bayesian Information Criterion (BIC) using a two-dimensional grid search. These two penalties aim to shrink the “irrelevant” regression coefficients towards zero (Fan and Peng 2004). Note that some epigenomic studies have stopped at this step (e.g., Yoon et al. 2017), i.e., after the regularization method is performed, with authors reporting the estimated “true” (i.e., non-zero) $\hat{\beta _k}$’s. Again, here, we are not advocating the Elastic-Net algorithm. The main contribution of our approach is delineating the steps needed to provide a valid and possibly-informative Fisher-exact *p*-value, which does not rely on asymptotic arguments. Future work should perform a simulation study varying, for example, the distribution of the high-dimensional potential outcomes, sample size, test statistic, and include power considerations.

**Step 2**: With the selected $\hat{\beta _k}$’s from Step 1, we construct the following scalar test statistic,

$$\begin{aligned} T=\sqrt{ \sum \limits _{k=1}^{K}\hat{\beta _k}^2}, \end{aligned}$$

which should be sensitive to the number of selected CpG sites and their effect sizes. There are many ways of constructing a test statistic in such a big data setting. To obtain a small-sample valid Fisher-exact *p*-value, any strategy needs to summarize high-dimensional empirical evidence by a *scalar*.

### Randomization-based causal inference

The actual assignment mechanism could not be precisely recovered (Bind and Rubin 2020). Consequently, we assume a Bernoulli mechanism with probability 0.5, which leads to $2^{17}$ possible randomized allocations with our data. Using the Harvard FAS cluster, we repeat the two-step procedure just described $2^{17}$ times assuming the sharp null hypothesis $H_{00}$, and thereby construct the null randomization distribution of *T*. The Fisher-exact *p*-value is the proportion of values of *T* calculated under $H_{00}$ that are as extreme or more extreme than the observed value of *T*. Note that the statistic *T* varies across randomizations, because of the selected non-zero $\hat{\beta _k}$’s, their number, their values, and their corresponding CpG sites.

## Results

The Elastic-Net procedure selected thirteen CpG sites (see Table 3) with the actual data. Among these thirteen CpG sites, eight were related to the following functional genes: Nuclear Prelamin A Recognition Factor-Like (*NARFL*), Myeloid Leukemia Factor 2 (*MLF2*), Zinc Finger Protein 513 (*ZNF513*), Pyruvate Dehydrogenase Phosphatase Regulatory Subunit (*PDPR*), Zinc Finger Protein 318 (*ZNF318*), ATP-Binding Cassette, Sub-Family F (*GCN20*), Member 3 (*ABCF3*), Chromosome 7 Open Reading Frame 50 (*C7orf50*), and Chromosome 1 Open Reading Frame 198 (*C1orf198*).

Recall that such a listing is the typical conclusion in current publications (e.g., Suchting et al. 2019). The implicit conclusion appears to be that these sites could be further examined in future investigations.

**Table 3:** Epigenetic sites selected by the Elastic-Net procedure and observed mean methylation under clean air and ozone exposures

Selected CpG sites	Gene related	Mean ($N=17$) methylation under clean air exposure	Mean ($N=17$) methylation under ozone exposure
cg00605859	–	0.018	0.020
cg00936733	*NARFL*	0.868	0.839
cg02977706	*MLF2*	0.026	0.031
cg03431514	*ZNF513*	0.023	0.026
cg05690286	*PDPR*	0.023	0.028
cg07003920	–	0.013	0.015
cg10689147	*ZNF318*	0.032	0.038
cg10877294	*ABCF3*	0.017	0.020
cg15536489	*C7orf50*	0.968	0.973
cg21159689	*C1orf198*	0.965	0.973
cg21964391	–	0.801	0.839
cg25538664	–	0.012	0.014
cg26662410	–	0.025	0.030

**Fig. 5:**
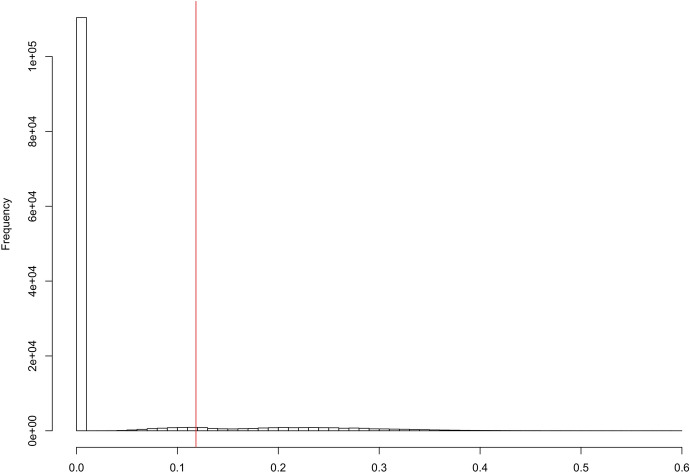
Null randomization distribution of T; the associated Fisher-exact *p*-value is $= \frac{16,203}{2^{17}} \approx 0.12$; 16,203 is the number of values of *T* in the null randomization distribution that are greater or equal to the observed value of *T* (displayed in red) (color figure online)

The distribution of the $2^{17}$ values of the test statistic T under the sharp null hypothesis is shown in Fig. 5, where the associated Fisher-exact *p*-value is $\frac{16,203}{2^{17}} \approx 0.12$; 16, 203 is the number of values of *T* that are greater or equal to the observed value of *T*.

## Discussion

### What if Fisher had access to high-performance computing?

This general procedure for a randomized experiment, we believe first described explicitly in Fisher (1925), is compatible with any test statistic and generates an associated valid *p*-value. To our knowledge, our initial version of this article is the first time that regularization methods are coupled with randomization-based inference to obtain a valid Fisher-exact *p*-value that does not rely on any asymptotic arguments. We speculate that randomization-based inference was not emphasized in Fisher’s earlier discussions because of the lack of high-performance computing during most of the $20{\text {th}}$ century. However, we now have computing capabilities to display the null randomization distributions, and we should do so to avoid relying on asymptotic approximations, which can be misleading (Bind and Rubin 2020).

### Display the null randomization distribution

Displaying a null randomization distribution may provide valuable scientific insights, as already argued by us (Bind and Rubin 2020). Here, we displayed a possibly-insightful null randomization distribution. First, this null randomization distribution has two major components: A point mass at zero corresponding to 110,403 allocations (i.e., $\approx \,84\%$), indicating that under the null hypothesis, most values of *T* would be zero, and the remaining randomized allocations that led to positive values of the test statistic with a maximum around 0.6. The location of the observed test statistic, T, is away from the point mass at zero but not near the most extreme null value of the test statistic. Again, future work should perform a simulation study varying, e.g., the high-dimensional potential outcomes, sample size, test statistic, and comparing characteristics of the resulting null randomization distributions.

### Selection methods

Here, we provide a non-parametric, non-asymptotic, approach to obtain a valid Fisher-exact *p*-value; in our context, this *p*-value was around 0.12, which suggests only quite modest evidence about any causal effects of ozone (vs. clean air) on DNA methylome. Although we chose to use the Elastic-Net procedure to construct our test statistic, other algorithms could have been used instead, such as the ones proposed by Li et al. (2015) (which selects non-zero $\hat{\beta }_k$ through “a penalized multivariate multiple linear regression model with an arbitrary group structure for the regression coefficient matrix”) or the one proposed by Cox and Battey (2017) (which capitalizes on partially balanced incomplete block designs to select several choices of sparse non-zero $\hat{\beta }_k$ that fit as well as the Lasso’s choice). Cox and Battey’s choices of sparse non-zero $\hat{\beta }_k$ would also need to be combined into a scalar test statistic. One idea would be to use a more general test statistic:

$$\begin{aligned} T=\sqrt{\max _{m} \sum \limits _{k=1}^{K}\hat{\beta }_{k,m}^2} \end{aligned}$$

where $\hat{\beta _{k,m}}$ represents the $k{\text {th}}$ coefficient for a model *m* that has essentially the same fit as the Lasso procedure.

### Limitations and strengths

Our example certainly has limitations. Our target experiment relying on seventeen human subjects with two outcome measurements as 34 units has low power because of the small number of units, even with no carry-over and no time effects between visits, assumptions which may not be plausible in our experiment. The literature is limited on both issues. Because the order of exposure is randomized, we expect important covariates (e.g., ambient air pollution exposure, dietary changes) to be balanced between groups, but this is not guaranteed. Regarding a potential carry-over effect of ozone on the DNA methylome, we believe this should be studied in animal and/or *in vitro* studies before conducting such crossover experiments with human participants. If this were the case, one should take into account the sequential feature of the crossover experiments and allow for the estimation of the carry-over effects, for example, using a model to estimate appropriate causal effects. However, for this effort to be helpful would require a larger number of participants than in this study.

Despite these limitations, however, we believe that this paper possesses some important messages. Because of the randomized treatment assignment induced by the study design, it is conceptually straightforward to implement a randomization-based test, and thanks to currently available high-performance computing, we can do this exactly. Also, if the “no carry-over and no time effects” assumptions hold, it would be natural to report that we found some weak evidence about an effect of ozone exposure on the DNA methylome.

### Computational considerations

For a single exposure allocation, the Elastic-Net procedure takes 100 seconds on a 2020 personal laptop, which would mean about 61 days for all $2^{17}$ allocations. Instead, we capitalized on parallel computing (i.e., 100 allocations per batch) available on the 2020 Harvard FAS cluster to construct the null randomization of the test statistic.

### Epilogue

Future directions on this topic include the extension of this procedure to non-randomized experiments. In this case, we would have to posit an assignment mechanism (Bind et al. 2014) and a class for sensitivity analysis (Bind and Rubin 2020); assuming the unconfoundedness assumption holds, the rest of the procedure is straightforward.

An alternative to the sharp null hypothesis testing is to use Bayesian model-based inference and explicitly impute the missing high-dimensional potential outcomes (Rubin 1978). The first step could be to model each potential outcome under clean air as mixtures of a finite number of distributions, and similarly for each potential outcome under ozone.

Here, using a significance level of 0.05, we would fail to reject the sharp null hypotheses stating that the effect of ozone vs. clean air on all 484,531 components of the epigenomic outcome is zero, but would not accept this sharp null hypothesis. We believe that it would be interesting to contemplate the multivariate counternull values (Rosenthal and Rubin 1994) (or sets) that, by definition, have the same randomization-based evidence as the null values we obtained here; this is a computational challenge that we hope to address.

## Data Availability

The high-dimensional data will not be deposited, but will be shared upon request.

## Footnotes

1 The 450K array covers 99% of the genes with an average of seventeen CpG sites per gene region distributed across the promoter, i.e., the region of DNA that initiates gene transcription. About 70% of promoters have high CpG content. Data were pre-processed with the beta-mixture quantile normalization method (BMIQ) method (Teschendorff et al. 2013). The result was, for each participant, a vector outcome of 484,531 measured proportions.

2 Note that in the more general randomized experiment where the assignment is randomized at both periods $j=1$ and $j=2$, the corresponding Fisher’s sharp null hypothesis would include two additional statements:

$$\begin{aligned} Y_{i,j=2}(W_{i,j=1}=0, W_{i,j=2}=1) = Y_{i,j=2}(W_{i,j=1}=0, W_{i,j=2}=0) \end{aligned}$$

and

$$\begin{aligned} Y_{i,j=2}(W_{i,j=1}=1, W_{i,j=2}=0) = Y_{i,j=2}(W_{i,j=1}=1, W_{i,j=2}=1). \end{aligned}$$

In the context of our randomized crossover experiment, these last two statements can be omitted, because they have no empirical consequences: neither $Y_{i,j=2}(W_{i,j=1}=0, W_{i,j=2}=0)$ nor $Y_{i,j=2}(W_{i,j=1}=1, W_{i,j=2}=1)$ are observed.

## References

[CR1] Bell ML, McDermott A, Zeger SL, Samet JM, Dominici F (2004). Ozone and short-term mortality in 95 US urban communities, 1987–2000. JAMA.

[CR2] Bennett MR, Hasty J (2007). A DNA methylation-based switch generates bistable gene expression. Nat Genet.

[CR3] Bind MC, Rubin DB (2020). When possible, report a Fisher-exact $P$ value and display its underlying null randomization distribution. Proc Natl Acad Sci USA.

[CR4] Bind MA, Lepeule J, Zanobetti A, Gasparrini A, Baccarelli A, Coull BA, Tarantini L, Vokonas PS, Koutrakis P, Schwartz J (2014). Air pollution and gene-specific methylation in the Normative Aging Study: association, effect modification, and mediation analysis. Epigenetics.

[CR5] Bind MC, Rubin DB, Cardenas A, Dhingra R, Ward-Caviness C, Liu Z, Mirowsky J, Schwartz JD, Diaz-Sanchez D, Devlin RB (2020). Heterogeneous ozone effects on the DNA methylome of bronchial cells observed in a crossover study. Sci Rep.

[CR6] Bonferroni C (1936). Teoria statistica delle classi e calcolo delle probabilita. Pubblicazioni del R Istituto Superiore di Scienze Economiche e Commerciali di Firenze.

[CR7] Conway DA, Roberts HV (1983). Reverse regression, fairness, and employment discrimination. J Bus Econ Stat.

[CR8] Cox DR, Battey HS (2017). Large numbers of explanatory variables, a semi-descriptive analysis. Proc Natl Acad Sci USA.

[CR9] Devlin RB, Duncan KE, Jardim M, Schmitt MT, Rappold AG, Diaz-Sanchez D (2012). Controlled exposure of healthy young volunteers to ozone causes cardiovascular effects. Circulation.

[CR10] Edgeworth FY (1885) Methods of statistics. J Stat Soc Lond 181–217. ISSN 09595341. http://www.jstor.org/stable/25163974

[CR11] Fan J, Peng H (2004) Nonconcave penalized likelihood with a diverging number of parameters. Ann Stat 32(3):928–961. ISSN 00905364. http://www.jstor.org/stable/3448580

[CR12] Fisher R (1925). Statistical methods for research workers.

[CR13] Friedman J, Hastie T, Tibshirani R (2010). Regularization paths for generalized linear models via coordinate descent. J Stat Softw.

[CR14] Holland PW (1986) Statistics and causal inference. J Am Stat Assoc 81(396):945–960. ISSN 01621459. http://www.jstor.org/stable/2289064

[CR15] Jerrett M, Burnett RT, Pope CA, Ito K, Thurston G, Krewski D, Shi Y, Calle E, Thun M (2009). Long-term ozone exposure and mortality. N Engl J Med.

[CR16] Li J, Li WX, Bai C, Song Y (2015). Particulate matter-induced epigenetic changes and lung cancer. Clin Respir J.

[CR17] Miller CN, Dye JA, Schladweiler MC, Richards JH, Ledbetter AD, Stewart EJ, Kodavanti UP (2018). Acute inhalation of ozone induces DNA methylation of apelin in lungs of Long-Evans rats. Inhal Toxicol.

[CR18] Morozova TV, Huang W, Pray VA, Whitham T, Anholt RR, Mackay TF (2015). Polymorphisms in early neurodevelopmental genes affect natural variation in alcohol sensitivity in adult drosophila. BMC Genomics.

[CR19] Neyman J (1923–1990) On the application of probability theory to agricultural experiments. Essay on principles. Section 9. translated in Statistical Science 5(4):465–472. 10.1214/ss/1177012031

[CR20] Perneger TV (1998). What’s wrong with Bonferroni adjustments. BMJ.

[CR21] Rosenthal R, Rubin DB (1994) The counternull value of an effect size: a new statistic. Psychol Sci 5(6):329–334. ISSN 09567976, 14679280. http://www.jstor.org/stable/40063131

[CR22] Rubin D (1974). Estimating causal effects of treatments in randomized and nonrandomized studies. J Educ Psychol.

[CR23] Rubin DB (1978). Bayesian inference for causal effects: the role of randomization. Ann Stat.

[CR24] Rubin DB (1980) “Comment” on “Randomization analysis of experimental data: the fisher randomization test” by Basu (TM). J Am Stat Assoc 75(369):591. ISSN 0162-1459

[CR25] Rubin DB (1990) [On the application of probability theory to agricultural experiments. Essay on principles. Section 9]. Comment: Neyman (1923) and Causal inference in experiments and observational studies. Stat Sci 5(4):472–480. 10.1214/ss/1177012032

[CR26] Suchting R, Hébert ET, Ma P, Kendzor DE, Businelle MS (2019) Using elastic net penalized cox proportional hazards regression to identify predictors of imminent smoking lapse. Nicotine Tobacco Res 21(2):173–179. ISSN 1462-2203 10.1093/ntr/ntx201 PMC7962780 29059349

[CR27] Teschendorff AE, Marabita F, Lechner M, Bartlett T, Tegner J, Gomez-Cabrero D, Beck S (2013). A beta-mixture quantile normalization method for correcting probe design bias in Illumina Infinium 450 k DNA methylation data. Bioinformatics.

[CR28] Turner MC, Jerrett M, Pope CA, Krewski D, Gapstur SM, Diver WR, Beckerman BS, Marshall JD, Su J, Crouse DL, Burnett RT (2016). Long-term ozone exposure and mortality in a large prospective study. Am J Respir Crit Care Med.

[CR29] Wasserstein RL, Schirm AL, Lazar NA (2019). Moving to a world beyond “0.05”. Am Stat.

[CR30] Yoon G, Zheng Y, Zhang Z, Zhang H, Gao T, Joyce B, Zhang W, Guan W, Baccarelli AA, Jiang W, Schwartz J, Vokonas PS, Hou L, Liu L (2017) Ultra-high dimensional variable selection with application to normative aging study: DNA methylation and metabolic syndrome. BMC Bioinform 18(1):156. ISSN 1471-2105 10.1186/s12859-017-1568-1 PMC5340011 28264653

[CR31] Zeilinger S, Kuhnel B, Klopp N, Baurecht H, Kleinschmidt A, Gieger C, Weidinger S, Lattka E, Adamski J, Peters A, Strauch K, Waldenberger M, Illig T (2013). Tobacco smoking leads to extensive genome-wide changes in DNA methylation. PLoS ONE.

[CR32] Zhong J, Karlsson O, Wang G, Li J, Guo Y, Lin X, Zemplenyi M, Sanchez-Guerra M, Trevisi L, Urch B, Speck M, Liang L, Coull BA, Koutrakis P, Silverman F, Gold DR, Wu T, Baccarelli AA (2017). B vitamins attenuate the epigenetic effects of ambient fine particles in a pilot human intervention trial. Proc Natl Acad Sci USA.

